# Fabrication of Flexible Multilayer Composite Capacitors Using Inkjet Printing

**DOI:** 10.3390/nano10112302

**Published:** 2020-11-20

**Authors:** Timo Reinheimer, Viktoria Baumann, Joachim R. Binder

**Affiliations:** Institute for Applied Materials, Karlsruhe Institute of Technology, Hermann-von-Helmholtz-Platz 1, 76344 Eggenstein-Leopoldshafen, Germany; viktoria_baumann@web.de (V.B.); joachim.binder@kit.edu (J.R.B.)

**Keywords:** inkjet printing, printed capacitors, ceramic/polymer composites, multilayer

## Abstract

This paper shows a straightforward method for printing multilayer composite capacitors with three dielectric layers on flexible substrates. As known from multilayer ceramic chip capacitors (MLCCs), it is possible to create a parallel connection of the layers without enlarging the needed area. Hence, the overall capacitance is increased, as the capacitances of the single dielectric layers add up. To realize printed capacitors, a special ceramic/polymer composite ink is used. The ink consists of surface-modified Ba_0.6_Sr_0.4_TiO_3_ (BST), a polymeric crosslinking agent and a thermal initiator, which allows an immediate polymerization of the ink, leading to very homogenous layers. The dielectric behavior of the capacitors is examined for each completed dielectric layer (via impedance spectroscopy) so that the changes with every following layer can be analyzed. It is demonstrated that the concept works, and capacitors with up to 3420 pF were realized (permittivity of ~40). However, it was also shown that the biggest challenge is the printing of the needed silver electrodes. They show a strong coffee stain effect, leading to thicker edge areas, which are difficult to overprint. Only with the help of printed supporting structures was it possible to lower the failure rate when printing thin dielectric layers.

## 1. Introduction

Modern electronic components are increasingly emerging from the field of printed electronics, whose market is predicted to have enormous potential in the coming years. The methods behind this trend are the low cost and low complexity, which makes them very attractive [1].

An integral part of a large number of electrical components is capacitors, which are the most important components for energy storage in electronic circuit technology [2]. A consequential step is, therefore, the development of fully printed, flexible capacitors. At present, the dielectric layers of such capacitors are mainly made of polymeric materials [3,4], which means that their capacitances are rather low. In order to develop high-performance components, it is necessary to maximize capacitance without losing flexibility. On the one hand, this can be achieved through the combined use of ceramics and polymers for the dielectric layer because ceramics have a much higher dielectric constant and thus increase the capacitance [5,6,7,8]. On the other hand, the production of extremely thin layers is advantageous because the capacitance is also increased by a smaller distance of the electrodes [9]. In order to achieve these goals, inkjet printing as an additive manufacturing process has proven to be eminently suitable [4,10]. The use of particulate inks is possible, and the requirements for a general printability of the ink allows a high dilution. With appropriate ink development, it is possible to produce thin layers of under one micron.

Another promising idea is to print multilayer capacitors with more than one dielectric layer. With a suitable print layout, a parallel connection of the dielectric layers can be created, whereby the capacitances of the individual layers add up; thus, an increase in the overall capacitance is achieved, while the effective capacitor area remains the same. This concept is already used for multilayer ceramic chip capacitors (MLCCs), which can be manufactured in different ways. A major challenge here is the processing of the ceramic particles in order to obtain the thinnest possible layers. Inkjet printing as an additive manufacturing process is very suitable for this, as has already been shown [11,12,13].

This paper aims to print flexible multilayer capacitors, with the use of a composite ink and a polymer substrate. The concept will be demonstrated with the help of capacitors with three parallel-connected dielectric layers, printed with different drop spacing. For that, a ceramic/polymer composite ink is used, which consists of surface-modified Ba_0.6_Sr_0.4_TiO_3_ (BST) particles and poly(ethylene glycol) diacrylate (PEG-DA). This special polymerizable ink system was successfully used before, and it results in very thin and homogenous dielectric layers [9].

In order to assess the performance of the printed multilayer capacitors, the capacitance *C* and the loss factor are measured via impedance spectroscopy. The microstructure and the layer thickness *d* are determined via scanning electron microscope (SEM) images, so that the relative permittivity of the dielectric *ε*_r_ can be calculated with the following formula:

(1) $$\varepsilon_{r}=\frac{C\times d}{\varepsilon_{0}\times A}$$

where *ε*_0_ is the dielectric constant, and *A* is the effective capacitor area. For connecting the permittivity of the layers in parallel, the layer thickness must be calculated as the reciprocal of the sum of the reciprocal individual values:

(2) $$d=\frac{1}{\frac{1}{d_{1}}+\frac{1}{d_{2}}+\ldots +\frac{1}{d_{n}}}$$

## 2. Materials and Methods

As crosslinking agent, poly(ethylene glycol) diacrylate (PEG-DA, M_n_: 700, Sigma Aldrich, St. Louis, MO, USA) was used. 3-(Trimethoxysilyl)propyl methacrylate (TMSPMA, Sigma Aldrich, St. Louis, MO, USA) was used for the surface modification. To stabilize the ceramic dispersion, DOLACOL D1001 (Zschimmer & Schwarz, Lahnstein, Germany) was added. Dimethyl 2,2’-azobis(2-methylpropionate) (Wako Chemicals, Neuss, Germany) was used in the reactive ink as a thermal radical initiator.

### 2.1. BST Synthesis

BST was produced using a sol-gel method. A 6 L reactor was flushed with nitrogen, and acetic acid (30.000 mol), barium acetate (0.422 mol) and strontium acetate hemihydrate (0.281 mol) were added under stirring. After the powder was well dispersed, titanium isopropoxide (0.703 mol) was added and stirred again. Water (181.800 mol) was added to the sol, and the mixture was filtered (polytetrafluoroethylene, 1 µm) and spray-dried (MM-HT-ex laboratory spray dryer, Niro, Søborg, Denmark). The precursor was calcined at 1100 °C for two hours in a tube furnace (CTF1600; Heraeus, Hanau, Germany). To obtain a high particle surface for the modification in the next step, the powder was milled in a stirred media mill (MiniCer, Netzsch, Selb, Germany) using 200 µm ZrO_2_ milling beads.

### 2.2. Surface Modification

The nanoparticles (<200 nm) were modified similarly to a method of Xie et al. [14]. First, BST (0.235 mol) was hydroxylated with H_2_O_2_ (2.449 mol; 30 wt%) at 60 °C. The reaction dispersion was stirred overnight, and the obtained powder (BST-OH) was separated by centrifugation. The powder was washed with water (3.333 mol) and dried in a vacuum oven at 80 °C.

BST-OH (0.188 mol) was then modified with TMSPMA (0.016 mol) in dried toluene (3.022 mol) under an argon atmosphere. The reaction dispersion was stirred overnight at 100 °C, and the powder (BST-Si) was again separated by centrifugation, washed with toluene (0.755 mol) and dried in a vacuum oven at 80 °C.

### 2.3. Ink Preparation

To achieve a stable ceramic dispersion, BST-Si was milled again in the stirred media mill, resulting in a particle size distribution of 39–197 nm (median: 72 nm). The used amounts resulted in an 8.5 vol% dispersion of BST-Si in butyl diglycol (BDG), which was stabilized with the dispersant (1.5 vol%). To obtain the print-ready ink, the dispersion was mixed with a solution of PEG-DA in isopropanol (IPA) (20 vol%), resulting in a volume ratio of BST-Si to PEG-DA of 50:50. Finally, the initiator was added (1 mg/mL), and the ink was diluted with BDG to have an overall solid content of 10 vol%.

### 2.4. Inkjet Printing

The used inkjet printer (Autodrop Professional; Microdrop, Norderstedt, Germany) with a Drop-on-Demand system was equipped with a single nozzle with a diameter of 70 µm for the dielectric ink. The used drop spacing was between 70 and 90 µm and is mentioned in the results. The ink vessel was set under a vacuum of −10 mbar. The droplets were generated by applying a negative voltage of −90 V and a pulse length of 32 µs at a frequency of 500 Hz. The printhead was heated to 25 °C, while the temperature of the substrate table was set to 70 °C. The composite areas of 5 × 5 mm^2^ were printed on untreated polyethylene terephthalate (PET) substrates (Melinex ST506, *d* = 175 μm, DuPont, Wilmington, DE, USA). The electrodes (2 × 8 mm^2^) were printed with a silver ink (Silverjet DGP-40LT-15C, Aldrich, St- Louis, MO, USA) using a 100 µm nozzle at 80 °C with a drop spacing of 130 µm for the bottom electrode and 100 µm for every following electrode. Here, a negative voltage of −120 V and a pulse length of 32 µs at a frequency of 500 Hz were used. The effective area of the capacitors was 4 mm^2^.

### 2.5. Characterization

SEM: To analyze the printed capacitors, the component was cut through in the middle, and an ion beam slope cutter (Leica EM TIC 3X, Leica Microsystems, Germany) was used to obtain a clearly recognizable cross-section. Images were then taken by SEM (Supra 55, Carl Zeiss, Oberkochen, Germany) with an AsB detector.

3D-topographies: The 3D-structures of the electrode and a printed capacitor were created with a chromatic white light interferometer (MicroProf; Fries Research & Technology, Bergisch Gladbach, Germany). The lateral resolution was set to 10 µm. The surface roughness in the center area of 4 × 4 mm^2^ was determined via the software FRT Mark III in accordance with DIN EN ISO 4287.

Dielectric properties: The capacitance and the loss factor were determined via an impedance analyzer (E4980AL, Keysight Technologies GmbH, Böblingen, Germany) using a voltage amplitude of 1 V and a frequency of 200 kHz.

## 3. Results and Discussion

**Figure 1:**
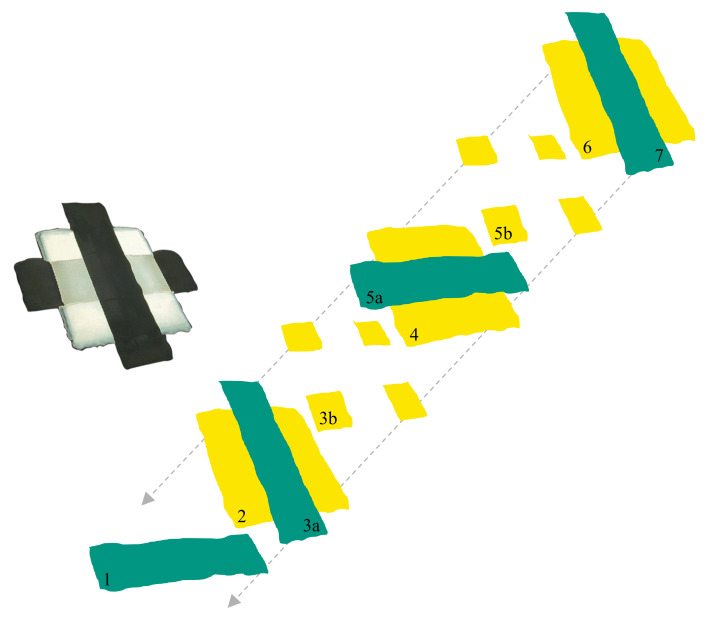
Microscope image (left) and schematic diagram (right) of a multilayer capacitor with three dielectric layers (yellow) and four Ag-electrodes (green). (1) First electrode; (2) First dielectric layer; (3a) Second electrode; (3b) Supporting structure; (4) Second dielectric layer; (5a) Third electrode; (5b) Supporting structure; (6) Third dielectric layer; (7) Fourth electrode.

To achieve a parallel connection of the composite layers, the print layout must be chosen accordingly. Here, three stacked composite layers should be implemented, separated by silver electrodes as is shown in Figure 1. The electrodes were printed over cross so that only every second electrode connected to one another (Figure 1: 1 and 5a, 3a and 7), resulting in an effective capacitor area of 4 mm^2^. 

**Figure 2:**
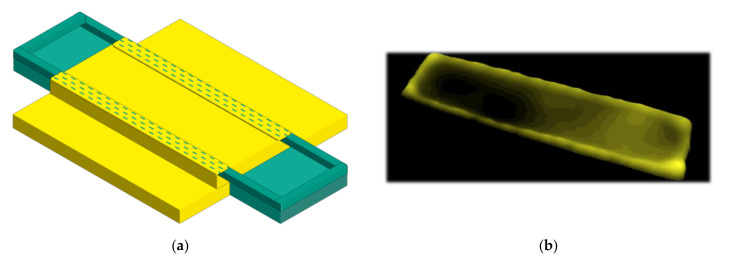
(**a**) Schematic representation of a printed composite layer on a silver electrode, which has a thick edge area. The dashed area of the composite layer represents the thinnest part, which is susceptible to cracking; (**b**) 3D-topography of a printed silver electrode on PET, with the intended thick edge area.

For printing the composite, a polymerizable ceramic ink system was used (Figure 1: 2, 4 and 6). The print-ready ink consisted of 5 vol% BST-Si, 5 vol% PEG-DA, an azo-initiator, IPA and BDG. The polymerizability and the drying behavior were examined. It was shown that the successful surface modification led to a fast increase in viscosity directly after the ink drop contacted the heated substrate; therefore, the modification led to very homogenous layers, even when printing low layer thicknesses. Details concerning the production and the behavior of the ink can be found in our previous work [9]. To obtain homogenous structures for every single layer, it was necessary to print supporting structures (Figure 1: 3b and 5b) for the composite layers, excluding the first layer. The electrodes showed a thick edge area that was difficult to overprint. The printed composite layer on top of this electrode then became much thinner at the edge area, as is illustrated in the image aside (Figure 2a). The thickness of the supporting structures was adapted to the thickness of the edge area of the electrodes, which helped the next printed layer become dense and homogenous. The four quadrates were printed with a drop spacing of 100 µm each, which resulted in a layer thickness of about 700 nm. To show the clearly-thicker edge area of the electrodes, an image of the 3D-topography of a printed electrode on PET is shown in Figure 2b. When printing the first layer, this unevenness can be compensated quite well, but for every following layer the supporting structures are essential. Printing without these structures can lead to a short circuit when printing the third electrode.

**Figure 3:**
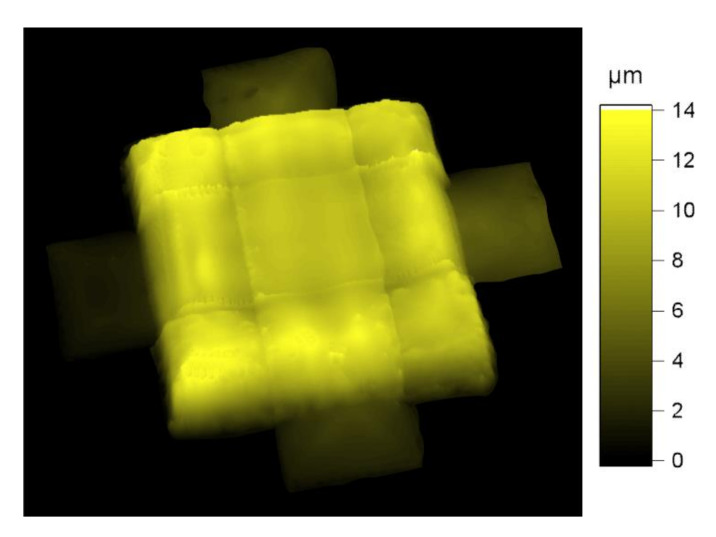
3D-topography of a printed multilayer capacitor, with three parallel-connected dielectric layers (each printed with a drop spacing of *p* = 90 µm), printed as shown in Figure 1.

This concept seemed to work quite well, as can be seen with the help of the 3D-topography of a printed multilayer capacitor with three parallel-connected dielectric layers (Figure 3). The most important part in the middle of the capacitor was very even and showed no inhomogeneities (surface roughness 185 nm). The entire outer area, around the effective capacitor area, became evenly thicker towards the edge. Details on this will be given later with the help of SEM images.

To investigate the dielectric behavior of the multilayer capacitors, two different combinations of drop spacing for the dielectric layers were used, with *p* = 70-70-70 and 70-80-80 µm. The layer thicknesses were determined via SEM images, and the capacitances as well as the loss factors were measured via impedance spectroscopy at a frequency of 200 kHz. As additional supporting structures are printed, the same drop spacing can lead to thicker layers for the second and the third composite layer. In order to examine the dielectric properties of the multilayer capacitors successively, the capacitance and the loss factor were measured after each completed dielectric layer, which was equipped with an electrode. After every printed layer, the structures were dried at 90 °C in a vacuum furnace for 20 h so that the conductivity of the silver electrodes was high enough (resistivity < 10 Ω). First, the capacitors with a drop spacing of each 70 µm were studied, for which the dielectric properties for every additional layer are shown in Table 1.

**Figure 4:**
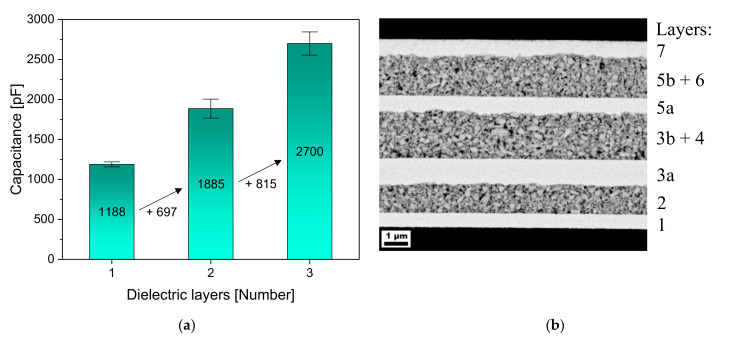
(**a**) Increasing capacitances of the multilayer capacitors with every additional layer, printed with a drop spacing of each 70 µm; (**b**) SEM image of the corresponding three-layered capacitor, with the same labeling as in Figure 1. (1) First electrode; (2) First dielectric layer; (3a) Second electrode; (3b) Supporting structure; (4) Second dielectric layer; (5a) Third electrode; (5b) Supporting structure; (6) Third dielectric layer; (7) Fourth electrode.

The layer thicknesses increased, as is to be expected based on the used supporting structures for the second and the third layer. The second layer became the thickest with 2.0 μm, while the first layer was 1.2 µm and the third layer was 1.8 µm thick. The number of pores remained roughly the same for each layer (0.8–0.9%) so that this could not have caused any change in layer thickness. The fact that the third layer was thinner than the second can be due to the ink running more strongly towards the edge, which can be caused by a gradient in the overall thickness of the capacitor. The edge area will be examined separately for this. The capacitance for the first layer was determined based on 15 samples and showed a high uniformity, similar to the loss factor. The number of samples for every additional layer represents the failure rate. After adding the second and the third layers, the values varied a little more, especially for the loss factors. There were samples that still showed an equal loss factor to before, but there were also samples that had approximately twice the loss factor. In principle, a constant loss factor is plausible because a parallel connection does not lead to higher losses, which are mainly caused by heat loss of the dielectric [2,15]. The SEM image of a three-layer capacitor shows that all layers could be added together perfectly, and no defects could be seen at the boundary layers (Figure 4b). The increase in capacitance with each additional layer is shown graphically in the adjacent figure (Figure 4a).

In comparison to the single-layer capacitor, the capacitance increased by 697 and then 815 pF, which corresponds to a total capacitance of 2700 pF. The approach of realizing a multilayer capacitor to increase the capacitance consequently worked very well, although each layer was thicker than in the case of a possible single-layer capacitor. In our previous work, we showed a single-layer capacitor with a thickness of the dielectric of only 700 nm, which had a capacitance of 2000 pF [9].

**Figure 5:**
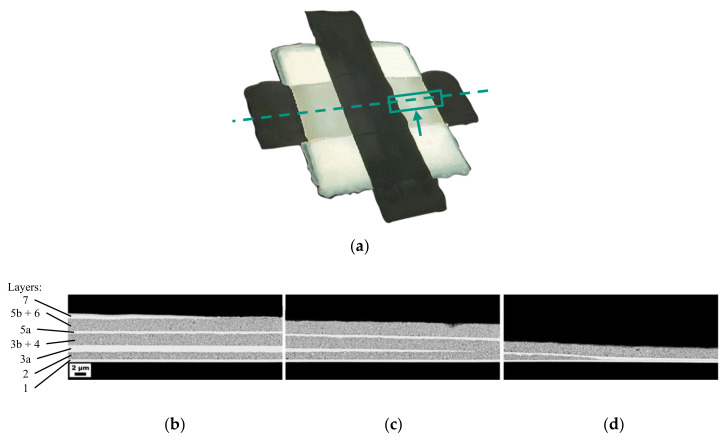
(**a**) Microscope image in which the dashed line shows the cut made to prepare the SEM images. The rectangle represents the area shown in the following cross-sectional SEM images, and the arrow shows the viewing direction; (**b**–**d**) SEM images of the area shown in (a) with the same labeling as in Figure 1. (1) First electrode; (2) First dielectric layer; (3a) Second electrode; (3b) Supporting structure; (4) Second dielectric layer; (5a) Third electrode; (5b) Supporting structure; (6) Third dielectric layer; (7) Fourth electrode.

In order to show the implemented structure of the three parallel-connected capacitor layers, the edge area of the capacitor is shown in Figure 5. The capacitor was cut along the dashed line (Figure 5a), and the following SEM images show the marked area of the capacitor. The beginning of the third electrode, which contacts the first electrode (see Figure 1: 1, 5a), can be seen in Figure 5d. The layer thickness between the first and the third electrode then increased continuously, and the beginning of the second electrode could be seen (Figure 5c). The fourth electrode should theoretically start at the same point (see Figure 1: 3a, 7), but started about 200 µm further on (Figure 5b). However, this small deviation did not have a major influence on the measured dielectric properties. It is probably caused by a slightly differently placed substrate on the heating plate. In addition, the top composite layer became very thick at the edge. This means the composite ink in the third layer ran through a steeper gradient to the edge and, thus, created a slightly thinner layer in the middle compared to the second layer with the same drop spacing.

Next, multilayer capacitors with a drop spacing of 70-80-80 µm were printed and analyzed as before. The failure rate was now significantly higher, to where only a single capacitor with three intact layers was realized. The dielectric properties of the capacitors for each additional layer are shown in Table 2. Despite the greater drop spacing from the second layer onwards, the second layer became thicker than the first due to the supporting structure. The third layer became thinner and had the smallest thickness of 1.2 µm. This tendency in the resulting layer thicknesses was also observed in the previously printed capacitors. The supporting structures thus ensured a thicker and more even composite layer, but the support already seemed to work less well with the third layer. The third layer was thinner than the second one, and there was a high failure rate after printing the third layer.

**Figure 6:**
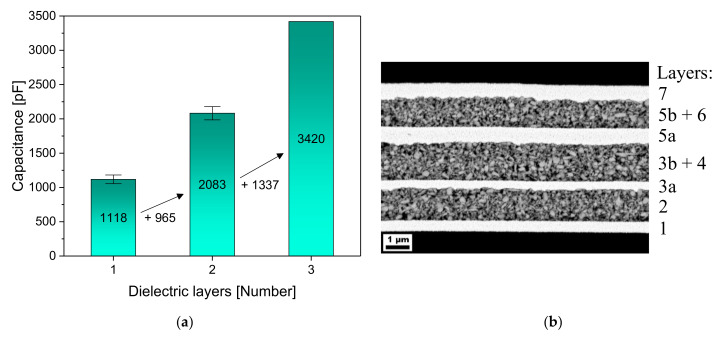
(**a**) Increasing capacitances of the multilayer capacitors with every additional layer, printed with drop spacings of 70-80-80 µm; (**b**) SEM image of the corresponding three-layered capacitor, with the same labeling as in Figure 1. (1) First electrode; (2) First dielectric layer; (3a) Second electrode; (3b) Supporting structure; (4) Second dielectric layer; (5a) Third electrode; (5b) Supporting structure; (6) Third dielectric layer; (7) Fourth electrode.

The three-layered capacitor showed a very high capacitance of 3420 pF and still had a comparable loss factor of 0.15. The increase in the capacitance for every additional layer is shown in Figure 6 with the corresponding SEM image. Even with two layers, a capacitance of over 2000 pF was achieved, which roughly corresponds to a single-layer capacitor with a layer thickness of only 700 nm. The layers in the SEM image also seem to complement each other very well, which raises the question of where the high failure rate comes from. A punctual crack at the edge area of the electrodes is most likely, as was discussed above with the help of Figure 2. In order to avoid this high failure rate, it is essential to develop silver inks that show no, or at least a significantly weaker, CSE. Thereby, it would be possible to print thinner dielectric layers and even more layers, which would further increase the capacitance.

## 4. Conclusions

The potential of functional multilayer printing was successfully demonstrated in this study. The developed layout allows the printing of a three-dimensional capacitor, consisting of three dielectric layers separated by silver electrodes. Thus, the required area for the capacitors is the same as for single-layer capacitors. The capacitance can therefore be increased, as the capacitances of the single layers add up. For example, with slightly different layer thicknesses for the three dielectric layers, the overall capacitance increased from 1118 to 2083 and finally to 3420 pF. The loss factor of this capacitor was 0.1447, and the used composite showed a permittivity of around 40 (at 200 kHz).

Essential for the success in printing dense structures for every dielectric layer was the printing of supporting structures. These structures are necessary to compensate the unevenness of the printed electrodes. Silver inks show a strong coffee stain effect, which leads to a thicker border area. This area is difficult to overprint, so the printed supporting structures have the same thickness and help the dielectric layer in being dense and homogenous. This concept works quite well in the case of three dielectric layers. However, it was shown that the failure rate increased with every additional dielectric layer, and the supporting structures’ influence on the homogeneity decreased. Thus, the development of silver inks that have no, or a clearly weaker, coffee stain effect is required. Realizing homogenous electrodes would help in printing thinner dielectric layers and in increasing the number of possible layers. In addition, the print design plays an important role in reducing the failure rate and should further be improved.

## Figures and Tables

**Table 1:** Capacitances, loss factors, permittivities and layer thicknesses of the multilayer capacitors with a drop spacing of each 70 µm. The measurements were carried out at 200 kHz and are averaged over 5 individual values for every single sample.

Drop Spacing [µm]	Capacitance [pF]	Loss Factor	Permittivity	Layer Thickness [µm]	No. of Samples
70	1188 ± 31	0.12 ± 0.01	40 ± 1	1,2	15
70-70	1885 ± 119	0.15 ± 0.06	40 ± 3	1.2/2.0	12
70-70-70	2700 ± 145	0.18 ± 0.03	40 ± 2	1.2/2.0/1.8	9

**Table 2:** Capacitances, loss factors, permittivities and layer thicknesses of the multilayer capacitors with a drop spacing of 70-80-80 µm. The measurements were carried out at 200 kHz and are averaged over 5 individual values for every single sample.

Drop Spacing [µm]	Capacitance [pF]	Loss Factor	Permittivity	Layer Thickness [µm]	No. of Samples
70	1118 ± 64	0.12 ± 0.01	41 ± 2	1.3	11
70-80	2083 ± 97	0.08 ± 0.003	41 ± 2	1.3/1.5	9
70-80-80	3420	0.15	43	1.3/1.5/1.2	1
